# Rare Lyme Carditis Presenting With Complete Heart Block in a Young Adult

**DOI:** 10.7759/cureus.105389

**Published:** 2026-03-17

**Authors:** Sara M Farah, Leen M Ahmed, Layan Alali, Majd Hamzeh, Ahmed M Saleh

**Affiliations:** 1 Faculty of Medicine, Hashemite University, Zarqa, JOR; 2 Internal Medicine, Henry Ford Health System, Rochester Hills, USA; 3 Internal Medicine, Henry Ford Rochester Hospital, Rochester Hills, USA; 4 Internal Medicine, Wayne State University School of Medicine, Detroit, USA

**Keywords:** complete heart block, lyme carditis, lyme disease, temporary pacemaker, third-degree atrioventricular block

## Abstract

Lyme carditis is a rare, yet highly treatable, manifestation of early disseminated Lyme disease, most commonly presenting as varying degrees of atrioventricular block. We report the case of a 27-year-old male who presented to the emergency department in Michigan, USA, on July 16, complaining of orthostatic dizziness and right-sided upper back pain. An electrocardiogram revealed third-degree atrioventricular block, requiring immediate temporary transvenous pacing and hospitalization. Intravenous ceftriaxone was then initiated. The patient’s heart block progressively resolved; the temporary pacemaker was removed on day five of admission, and he was discharged on day six to complete a 21-day course of intravenous antibiotics. Approximately two weeks prior to this visit, he had developed a severe headache, neck stiffness, and tingling in his fingers. He was evaluated in a primary care setting, where serological testing confirmed the diagnosis of Lyme disease, and he began oral doxycycline on July 12. Despite initial improvement in his headache, he presented to the emergency department with orthostatic dizziness and high-grade conduction delay. This case highlights the importance of recognizing subtle extracardiac symptoms of Lyme disease in endemic and at-risk areas to prevent unnecessary permanent pacemaker implantation.

## Introduction

Lyme disease, a spirochetal infection caused by species in the Borreliaceae family, stands as the most common vector-borne disease in the United States. Approximately 90% of all cases are reported from 15 high-incidence jurisdictions in the Northeast, Mid-Atlantic, and Upper Midwest regions. Signs and symptoms of early disease include erythema migrans (EM), a red, expanding rash often with central clearing, as well as fever and fatigue. An untreated infection can disseminate, affecting the heart, joints, and nervous system [1].

Among its various clinical manifestations, Lyme carditis emerges as a rare but potentially life-threatening complication of early disseminated Lyme disease, typically occurring within several days to about a month (average 21 days) after the initial illness/infection, most often in the summer and fall [2]. Affecting approximately 1% to 10% of diagnosed patients [3-4] and up to 11% of hospitalized Lyme disease patients [4], Lyme carditis predominantly impacts young adults, particularly young men [5-6].

The pathogenesis of Lyme carditis is thought to involve both direct spirochetal invasion and immune-mediated mechanisms. Immune-mediated injury has been implicated due to potential cross-reactivity between *Borrelia burgdorferi* antigens and cardiac epitopes, which may trigger lymphocytic infiltration of the cardiac tissue and conduction system. This inflammatory process can disrupt normal electrical conduction within the heart [7].

The hallmark of Lyme carditis is cardiac conduction system disease, most commonly presenting as varying degrees of atrioventricular (AV) block, which can rapidly progress to complete heart block [2,4]. While AV nodal block is the most frequent cardiac manifestation, other rare presentations include myocarditis, pericarditis, and endocarditis [2,4]. Patients may experience cardiac symptoms such as unexplained chest pain, bradycardia, or syncope [2-3,5]. Notably, these cardiac manifestations can occur even in the absence of the classic erythema migrans rash [8].

## Case presentation

A 27-year-old male presented to the Emergency Department complaining of dizziness and right-sided upper back pain, with vital signs notable for severe bradycardia and hypotension. Specifically, his heart rate was 20 beats per minute, blood pressure was 110/48 mmHg, respiratory rate was 22 breaths/min, oxygen saturation was 93% on room air, and his temperature was 37.5 °C (97.5°F).

**Figure 1:**
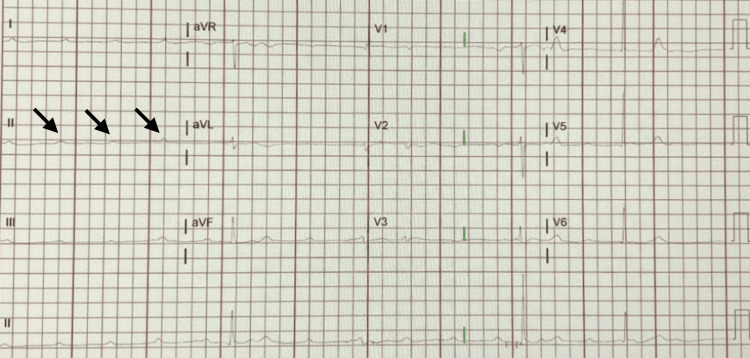
Twelve-lead electrocardiogram demonstrating complete heart block (third-degree atrioventricular block). Arrows (in lead II) highlight the P-waves marching independently of the QRS complexes, confirming total atrioventricular dissociation. A slow, narrow-complex junctional escape rhythm is observed at approximately 40 bpm.

Physical examination revealed an irregular bradycardic rhythm without jugular venous distention, heart murmurs, gallops, rubs, or peripheral edema. Skin turgor was normal, consistent with a euvolemic status. Lung auscultation revealed clear breath sounds bilaterally with no rales or wheezing. An electrocardiogram subsequently demonstrated marked sinus bradycardia and complete heart block, consistent with these clinical findings (Figure 1).

From June 29 to July 1, the patient was admitted to the hospital for evaluation of severe headaches, photosensitivity, neck stiffness, and tingling in his fingers. Laboratory evaluation revealed an elevated C-reactive protein level of 107 mg/L and leukocytosis, the latter attributed to recent steroid use. Blood cultures were negative, and magnetic resonance imaging of the brain and lumbar spine showed no enhancing lesions.

On July 11, he was evaluated in a primary care setting, where serologic testing confirmed IgM positivity for Lyme disease by enzyme-linked immunosorbent assay (ELISA) and Western blot. Treatment with oral doxycycline (100 mg twice daily) was initiated on July 12, and the patient reported improvement in his headache within 24 hours. Despite this initial improvement, he presented to the emergency department on July 16 with the previously noted symptoms.

Physical examination revealed no rash consistent with erythema migrans or other cutaneous manifestations of Lyme disease, and the patient denied recalling any such lesion during the current year. However, he reported playing disc golf outdoors approximately one month prior, suggesting potential tick exposure.

Echocardiography, consistent with his previously documented history of valvular dysfunction, demonstrated mild thickening of the mitral valve leaflet with mild regurgitation. Left ventricular cavity size and diastolic function parameters were within normal limits, with an estimated ejection fraction of 55-60%. A chest radiograph was unremarkable, with no evidence of pericardial effusion. Troponin I was 0.020 ng/mL.

**Figure 2:**
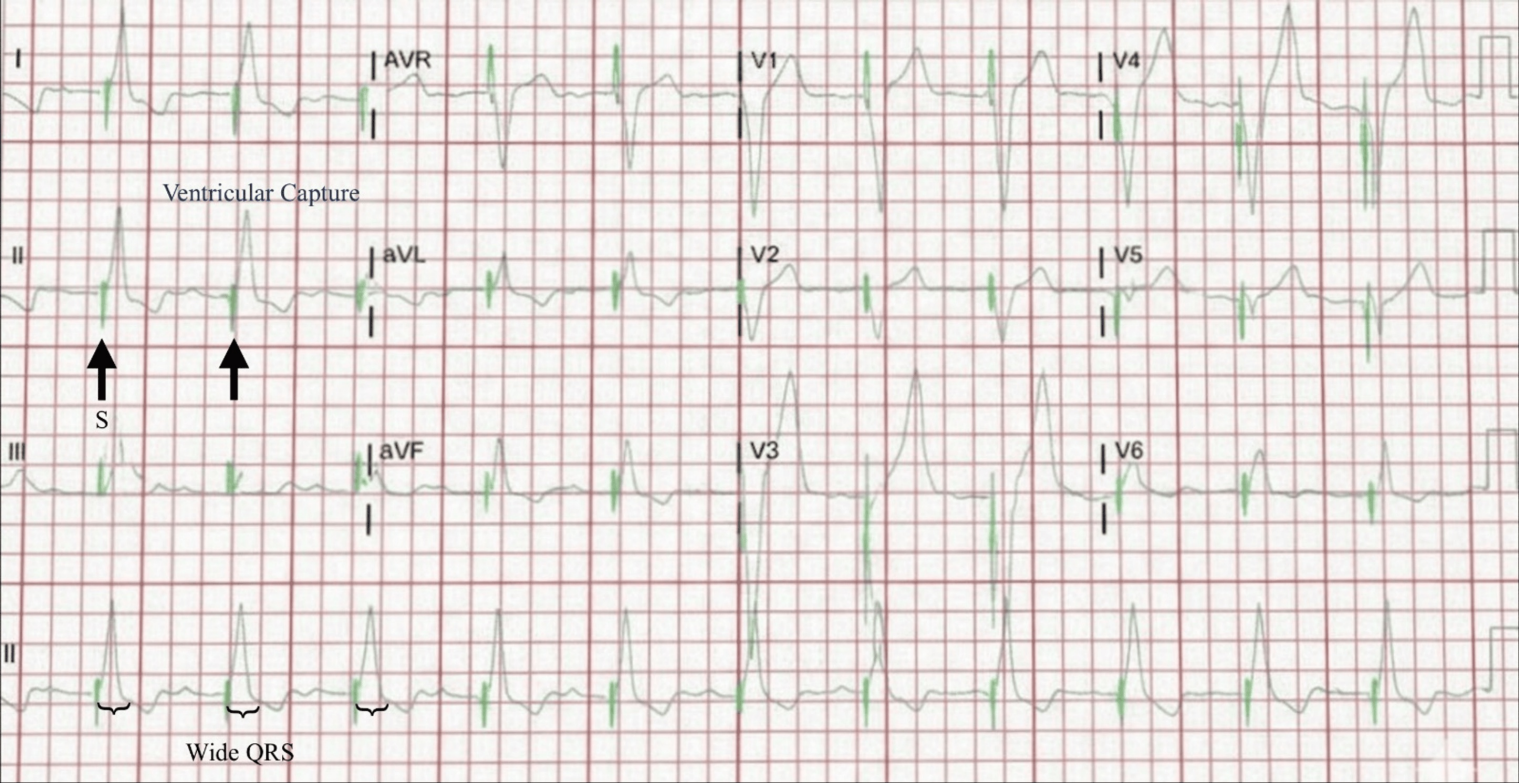
Twelve-lead electrocardiogram demonstrating a ventricular paced rhythm. Arrows indicate visible pacemaker spikes immediately preceding each wide QRS complex, confirming consistent ventricular capture.

Upon identification of third-degree AV block, the patient was promptly placed on a temporary transvenous pacemaker (Figure 2), and treatment was escalated to IV ceftriaxone 2g daily. Over the course of his hospital stay, his cardiac conduction abnormalities progressively resolved. By hospital day four, the pacing rate was successfully reduced to 40 bpm, at which point the patient consistently maintained stable intrinsic cardiac activity, indicating significant recovery of his conduction system. The temporary transvenous pacemaker was subsequently removed on day five. With complete resolution of the atrioventricular block confirmed by a final electrocardiogram (normal sinus rhythm, 62 bpm), the patient was discharged on hospital day six. Following discharge, he completed a 21-day course of intravenous ceftriaxone at an infusion center.

The patient's clinical progression and the specific management interventions used throughout his care are summarized in Table 1. To objectively illustrate the rapid resolution of his symptoms and the stabilization of his hemodynamic status following treatment escalation, a comparison of clinical parameters at admission and discharge is provided in Table 2.

**Table 1:** Clinical course and management timeline.

Day	Clinical phase	Clinical event and intervention
Day 14 (approximately)	Prodromal phase	Evaluated for severe headache, photosensitivity, and neck stiffness. Brain/spine MRI was normal (unremarkable).
Day 4 to Day 0	Initial diagnosis and outpatient Tx	Lyme IgM positive via serological testing. Initiated oral doxycycline (100 mg twice daily). Patient reported initial improvement in headache.
Day 0	Acute presentation and stabilization	Presented to ED with dizziness and back pain. EKG confirmed complete heart block (HR 20). Placed on temporary pacemaker and escalated to IV ceftriaxone (2 g daily).
Day 4	Conduction recovery	Pacing rate reduced; patient demonstrated consistent stable intrinsic cardiac activity (normal sinus rhythm, 62 bpm).
Day 6	Discharge	Discharged after confirming full recovery; transitioned to outpatient infusion center for completion of 21-day IV antibiotic course.

**Table 2:** Clinical and hemodynamic parameters at admission and discharge.

Parameter	Admission (July 16)	Discharge (July 21)
Heart rate (bpm)	20	62
Blood pressure (mmHg)	110/48	124/76
Rhythm	Complete heart block	Normal sinus rhythm
Symptoms	Dizziness, back pain	Asymptomatic
Support required	Transvenous pacing/IV	None (completed IV)

## Discussion

Managing Lyme carditis presents significant hurdles in both diagnostic confirmation and therapeutic escalation. Diagnosis is inherently complex, requiring a high index of suspicion and a combination of epidemiological data, clinical symptomatology, serology, and cardiac imaging to differentiate it from other inflammatory or infectious cardiomyopathies [9].

Our patient's presentation lacked the pathognomonic erythema migrans (EM) rash and instead presented with non-specific neurological symptoms (headache, stiffness, and paresthesias) approximately two weeks before presentation. This highlights the necessity of early serological testing when less common extracardiac symptoms appear in at-risk areas.

Critically, the patient's progression to third-degree AV block despite four days of oral doxycycline underscores the recognized temporal lag between the initiation of antimicrobial therapy and the resolution of the associated myocardial inflammatory infiltrate [7,10]. In accordance with the 2020 Infectious Diseases Society of America (IDSA)/American Academy of Neurology (AAN)/American College of Rheumatology (ACR) guidelines, symptomatic high-grade AV block necessitates hospital admission, continuous rhythm monitoring, and a transition to intravenous ceftriaxone [2]. While temporary pacing is reserved for cases of hemodynamic instability or high risk of asystole, its use in this patient was justified by his profound bradycardia and hypotension [2]. This aggressive intervention allowed for the successful stabilization of the patient and ultimately avoided the need for permanent pacemaker implantation [4].

## Conclusions

Lyme carditis warrants immediate consideration in the differential diagnosis for young, otherwise healthy patients presenting with unexplained bradycardia or high-grade atrioventricular (AV) block, particularly in endemic and tick-prevalent regions. As demonstrated by this case, where non-specific neurological symptoms preceded complete heart block without the characteristic erythema migrans rash, clinical suspicion must remain high. Crucially, the outcome confirms that prompt electrocardiographic diagnosis, followed by the timely initiation of a 21-day intravenous ceftriaxone and temporary cardiac pacing when needed, aligns with current clinical guideline recommendations. This aggressive, yet appropriate, intervention is the most effective way to ensure full cardiac conduction recovery and prevent the significant morbidity associated with unnecessary permanent pacemaker implantation.
